# Effect of aging and cementation systems on the bond strength to root dentin after fiber post cementation

**DOI:** 10.1590/0103-6440202305082

**Published:** 2023-03-06

**Authors:** Caio Henrique de Paula Nogueira, Mariana Bena Gelio, João Felipe Besegato, Anna Thereza Peroba Rezende Ramos, Eduardo Fernández, Milton Carlos Kuga, José Roberto Cury Saad

**Affiliations:** 1 Department of Restorative Dentistry, School of Dentistry, Araraquara, São Paulo State University - UNESP, Araraquara, São Paulo, Brazil; 2 Department of Restorative Dentistry, University of Chile, Santiago, Chile; 3 Institute of Biomedical Sciences, Autonomous University of Chile, Providencia, Chile.

**Keywords:** Dental cements, rhodamine B, confocal microscopy, resin cements, glass ionomer cement

## Abstract

This study evaluated the effect of aging and cementation of fiber posts using glass ionomer and resin cements on push-out bond strength, failure mode, and resin tag formation. One hundred and twenty bovine incisors were used. After post-space preparation, the specimens were randomly allocated into 12 groups (n = 10) according to the cementation system used: GC - GC Gold Label Luting & Lining); RL - RelyX Luting 2; MC - MaxCem Elite; RU - RelyX U200 and the aging periods (24 hours, 6 months, and 12 months). Slices from the cervical, middle, and apical thirds were obtained and analyzed by push-out bond strength test and confocal laser scanning microscopy. One-way ANOVA and Tukey’s post-hoc test was used at a significance level of 5%. For the push-out bond strength test, no differences among GC, RU, and MC in the cervical and middle thirds were observed, regardless of the period of storage (*P* > 0.05). In the apical third, GC and RU showed similar bond strength but higher than other groups (*P* > 0.05). After 12 months, GC showed the highest bond strength (*P* < 0.05). Bond strength to post-space dentin decreased over time, regardless of the cementation system used. Cohesive failure was the most frequent, regardless of the period of storage, cementation system, and post-space third. Tag formation was similar among all groups. After 12 months, GC showed the highest bond strength values.

## Introduction

Intra-radicular posts have been used to rehabilitate endodontically treated teeth with partial or total coronal destruction ^1^ ^,^ ^2^. The use of glass fiber posts (GFP) has increased compared to other intra-radicular posts ^3^ ^,^ ^4^ ^)^ due to their esthetic properties and elasticity modulus similar to dentin ^5^ ^,^ ^6^. These characteristics can reduce the risk of root fracture ^2^ by promoting homogenous dissipation of tensions among tooth, cement, and post ^7^.

Glass ionomer cements have been indicated as a luting system for metal-ceramic or metal-free prostheses, metallic posts, and GFP ^6^. Glass ionomer cements exhibit adequate chemical adhesion to dentin since its carboxyl groups bond to calcium ions from hydroxyapatite ^5^. In addition, the use of glass ionomer cements as a cementation system for GFP in endodontically treated teeth has shown promising results in terms of bond strength and dentin penetrability ^5^ ^,^ ^6^. 

Self-cure, light-cure, or dual-cure resin cements are routinely used for GFP cementation. One of the classifications of these cements is based on the bonding to dentin, which can be conventional or self-adhesive. Conventional resin cements are used after the application of adhesive system in the root canal, while self-adhesive cements do not require the use of adhesive systems due to its high chemical affinity with hydroxyapatite ^5^.

Self-adhesive resin cements have gained popularity ^8^ as a time-saving material, by reducing the technical sensitivity, making the cementation protocol easier and faster ^8^ ^,^ ^9^, and favoring the polymerization reaction in areas that light delivery is difficult ^5^. When using self-adhesive resin cement, the hybrid layer formation occurs over a dentin surface readily exposed and free of contaminates, which is ideal for bonding procedures ^10^. Particularly for dentin, the quality of bonding depends on the cement composition, bonding strategy used, and tissue characteristics ^4^.

The hybrid layer plays a crucial role in micromechanical retention ^11^, being expected that forms a stable and long-lasting bonding between dentin and resin cement ^12^. The hybrid layer formation consists of the infiltration of resin monomers into the collagen fibrils matrix exposed by acid demineralization, being directly related to the surface treatment of the dental tissue ^11^. Thus, the hybrid layer is an organic, hydrophobic, and acid-resistant interface. It has been reported that the use of self-adhesive resin cements promotes the hybrid layer formation over a dentin readily exposed and free of contaminates, which is ideal for bonding ^10^. However, regardless of the material or bonding strategy used, the hybrid layer is not always formed in a stable and homogenous manner ^12^, which may result in marginal infiltration, gap formation, and loss of retention ^12^ ^,^ ^13^.

Although the use of resin cements for GFP cementations has been extensively explored, there is a lack of studies evaluating glass ionomer cements. In addition, the majority of studies have demonstrated immediate results that do not represent the behavior of bonding over time. Thus, the bonding evaluation after extended periods is crucial to predict the material’s behavior and infer the possibility of a long-lasting treatment.

Herein, we aimed to evaluate the effect of aging (24 hours, 6 months, and 12 months) and type of cementation system (glass ionomer cement and self-adhesive resin cement) on the push-out bond strength to post-space radicular dentin after fiber post cementation. The null hypothesis tested was that there is no difference in the push-out bond strength regardless of the type of cementation system or period of storage.

## Material and methods

This experimental *in vitro* study received proper approval from the Ethical Committee in Animal Use of the School of Dentistry, Araraquara, São Paulo State University (UNESP), under the register number 1.603.859. The sample size was based on a pilot study and previously published studies ^5^ ^,^ ^14^.

### Specimens’ preparation

One hundred and twenty conoid bovine incisors with similar radicular anatomy dimensions were standardized based on radiographs taken in the buccolingual and mesiodistal directions. After extraction and selection, the teeth were in 0.1% thymol solution at 4ºC for 7 days. Afterward, the teeth were transversely sectioned using a precision cutting machine (IsoMet 1000; Buehler Ltd.) at 250 rpm and under constant water-cooling to obtain root specimens with 17 mm of length from the apex. Subsequently, the root canals were submitted to chemical-mechanical preparation and were dried with paper points and filled with the single cone technique using a F5 gutta-percha point (Dentsply Maillefer, Ballaigues, Switzerland) and AH Plus sealer (Dentsply DeTrey GmbH, Konstanz, Germany). After the vertical condensation, the cervical access of the specimens was sealed with glass ionomer cement (Maxxion R; FGM, Joinville, SC, Brazil) and kept in a relatively 99.9% humid environment at 37ºC for 7 days.

The post-space was prepared at 11 mm from the cervical root access using Largo burs #1 and #2 (Dentsply) and finalized with bur #2 (White Post DC; FGM). After that, the post-space was irrigated with 2.5% sodium hypochlorite (NaOCl) and dried with absorbent paper points. The surface of the GFP (DC #2 White Post; FGM) was cleaned with 95% ethanol solution (LabSynth). Sixty GFP, that were cemented with self-adhesive resin cement, were also etched with 37% phosphoric acid (Condac; FGM) for 1 minute, rinsed with distilled water, and dried with air-jet. After that, two layers of silane (Prosil; FGM) were applied over the whole surface of the fiber posts. In the remained sixty GFP, that were cemented with glass ionomer cement, no surface treatment was performed.

### Cementation protocols

After the proper surface treatment, the GFP were cemented with glass ionomer cement (GC - GC Gold Label Luting and Lining; and RL - Relyx Luting 2) (N = 30) or self-adhesive resin cement (MC - MaxCem Elite; and RU - RelyX U200) (N = 30). Box 1 displays the manufacturer, chemical composition, and cementation protocol used in this study. After the GFP cementation, the specimens of the groups MC - MaxCem Elite and RU - RelyX U200 were light-cured for 40 seconds with a LED unit (Valo; Ultradent Inc.) emitting an irradiance of 1000 mW/cm² that was positioned 1 mm from the surface of the fiber post. Additional light-curing was performed on each surface of the specimen (mesial, distal, buccal, and lingual) for also 40 seconds.

**Box 1:**
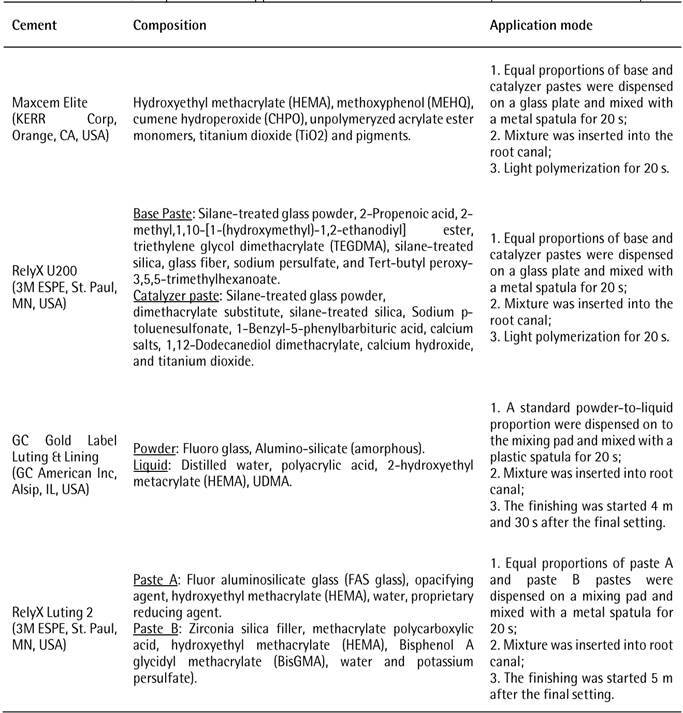
Trend name, composition and application mode of the cementation systems used in this study.

The dye rhodamine B (LabSynth) at 0.1% (% mass) was incorporated into the cementation systems of all specimens ^15^ ^,^ ^16^ ^,^ ^17^ to evaluate the tag formation in the post-space dentin. All clinical procedures were performed by only one properly trained operator.

### Push-out bond strength test

The specimens of each cementation system were divided into 3 groups (n = 10 per group) according to the period of storage (24 hours, 6 months, and 12 months). In the groups of 6 and 12 months, the specimens were kept immersed in distilled water at 37ºC, refreshing the medium every 2 days.

After the period of storage of each group, the roots were vertically placed inside of PVC matrices (21.3 of diameter x 20.0 mm of length) that was filled with polyester resin. One millimeter of the cervical root third was kept out of the inclusion. After 24 hours, the specimens were removed from the PVC matrices and sectioned perpendicularly to the long root axis to obtain slices (2.0 + 0.1 mm of depth) from the cervical, middle, and apical thirds of the post-space, which are respectively, 1 mm, 5mm, and 8mm of length from the cervical root access. The depth of each slice was verified with a digital pachymeter (Mitutoyo) and eventual irregularities were flatted with silicon carbide sandpapers (#1200; Norton).

The slices were carefully rinsed with distilled water, dried, and then submitted to the push-out bond strength test using a universal testing machine (EMIC) with a speed of 0.5 mm/min and load cell of 5 kN. To displace the set fiber post/cementation system, punch with 1.2 mm, 0.9 mm, and 0.5 mm of diameters were respectively used for the cervical, middle, and apical thirds of the post-space. The maximum force was obtained in newton (N) and then converted in megapascal (MPa) considering the adhesion area as described by Magro et al. ^18^

### Failure mode

After the push-out bond strength test, the cervical surface of each slice was analyzed in stereomicroscope (M125; Leica Microsystems) at x20 magnification to evaluate the incidence of failure mode, as described by Ramos et al. ^19^, in: type 1 (adhesive 1): when it occurred between the post and the cement; type 2 (adhesive 2): between dentin and cement; type 3 (cohesive): within the cement, and type 4 (mixed): when both types of failure were combined.

### Tag formation

One image of each post-space third was taken using a confocal laser scanning microscope (LSM 800 Airyscan; Carl Zeiss) at 10x magnification before the push-out test. All the images focused on the central region of the slice. The absorption and emission wavelength for rhodamine B were 540 nm and 494 nm, respectively. For each obtained image, forty mensuration (in µm) of the tag length were performed. These measurements were focused in the areas which had the most extended tags into the post-space dentin, using the Image J software (National Institutes of Health, Bethesda, Maryland, USA). The arithmetic mean of this forty mensuration was defined as the tag length for each slice analyzed. The image analyzes were evaluated by two independent evaluators (calibrated KAPPA= 0.85), where the groups evaluated were not known.

### Statistical analysis

Normal distribution and homoscedasticity of data from push-out bond strength and tag formation tests were verified by the Shapiro-Wilk test. One-way ANOVA and Tukey post-hoc tests were used for multiple comparisons. All the tests adopted a significance level of 5%. Failure mode data were presented in frequencies.

## Results

### Bond strength

**Table 1:** Mean and standard deviation of the bond strength values (MPa) at the post-space thirds according to the period of storage and the cementation systems.

Period of storage	Post-space thirds	Cementation systems			
		MC	RU	GC	RL
24 hours	cervical	13.55 + 0.96^aA^	14.26 + 2.72^aA^	15.11 + 1.61^aA^	9.59 + 0.88^bA^
	middle	12.31 + 1.54^aA^	13.18 + 1.99^aA^	13.86 + 1.68^aA^	9.75 + 0.62^bA^
	apical	9.94 + 0.99^bA^	13.01 + 0.94^aA^	13.85 + 1.09^aA^	9.30 + 0.76^bA^
6 months	cervical	13.26 + 1.29^aA^	13.56 + 1.18^aA^	15.01 + 1.25^aA^	9.39 + 0.69^bA^
	middle	12.26 + 0.86^aA^	12.98 + 0.85^aA^	13.79 + 1.21^aA^	9.65 + 1.05^bA^
	apical	9.56 + 0.71^bA^	12.95 + 1.11^aA^	13.74 + 1.49^aA^	9.21 + 0.77^bA^
12 months	cervical	8.26 + 1.27^cB^	10.06 + 1.59^bB^	13.71 + 1.51^aA^	7.69 + 1.08^cB^
	middle	7.66 + 0.72^cB^	9.68 + 1.89^bB^	13.48 + 1.17^aA^	7.35 + 0.94^cB^
	apical	7.66 + 1.19^cB^	9.11 + 0.95^bB^	13.24 + 1.03^aA^	7.11 + 0.98^cB^

a ^bc^ Different letters at the same row denote statistically significant difference according to the period of storage (p < 0.05). ^ABC^ Different letters at the same row denote statistically significant difference according to the cementation systems. Legends: MC - MaxCem Elite; RU - RelyX U200; GC - GC Gold Label Luting Lining; RL - RelyX Luting 2.

Table 1 displays the mean and standard deviation of bond strength values (MPa) of the cementation systems in each post-space third according to the period of storage and according the cementation system.

In the 24-hour and 6-month evaluation, no differences were found between GC (GC Gold Label Luting Lining), RU (RelyX U200), and MC (MaxCem Elite) at the cervical and middle thirds (*P* > 0.05). However, the bond strength of these groups was higher than RL (RelyX Luting) (*P* < 0.05). At the apical third, GC and RU showed similar bond strength (*P* > 0.05), but higher than MC and RL (*P* < 0.05).

In the 12-month evaluation, GC showed the highest bond strength regardless of the post-space third (*P* < 0.05). On the other hand, MC and RL showed the lowest bond strength (*P* < 0.05), but similar between them (*P* > 0.05).

All the cementation systems showed a bond strength decrease at the same third after 12 months of storage (*P* < 0.05), except for GC. However, no difference was found between 24 hours and 6 months of storage (*P* > 0.05).

### Failure mode

**Figure 1:**
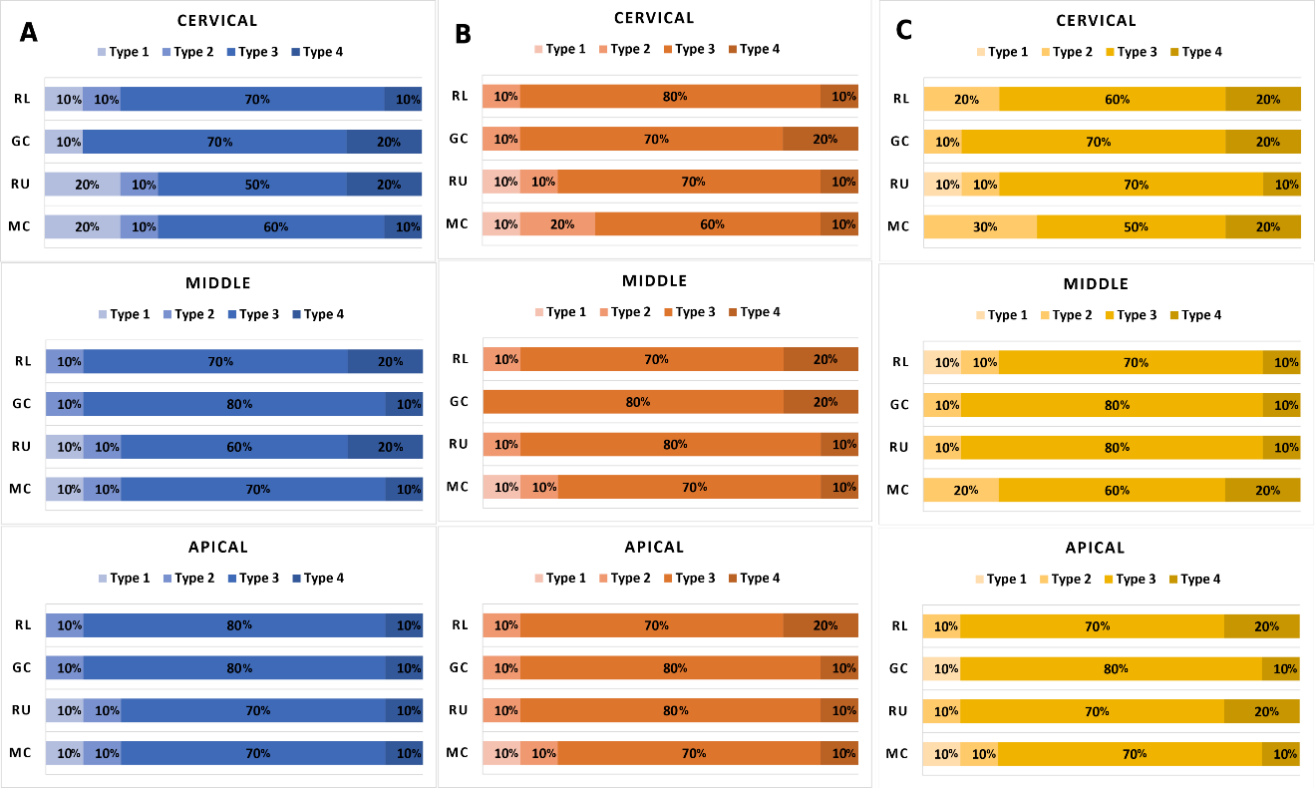
Percentual of failure mode for each group and post-space third after 24 hours (A), 6 months (B), and 12 months (C) of storage

Figure 1 shows the percentual of failure mode for each period of storage. Cohesive failure was the most frequent, regardless of the period of storage, cementation system, and post-space third.

### Tag formation

**Table 2:** Mean and standard deviation of the dentin penetrability (µm) into the dentin at each post-space third according to the cementation system and period of storage.

Cementation system	Post-space thirds	Period of storage		
		24 hours	6 months	12 months
MC	C	8.53 + 0.54	8.52 + 1.03	8.27 + 0.56
	M	8.49 + 0.31	8.34 + 0.49	8.26 + 0.51
	A	8.43 + 0.41	8.19 + 0.63	8.12 + 0.46
RU	C	8.53 + 0.48	8.69 + 0.91	8.46 + 0.51
	M	8.51 + 0.62	8.53 + 0.67	8.31 + 0.75
	A	8.44 + 0.59	8.16 + 0.63	8.18 + 0.55
GC	C	8.52 + 0.68	8.41 + 0.79	8.28 + 0.46
	M	8.46 + 0.54	8.25 + 0.51	8.27 + 0.35
	A	8.37 + 0.34	8.16 + 0.43	8.10 + 0.43
RL	C	8.61 + 0.77	8.77 + 0.73	8.50 + 0.47
	M	8.60 + 0.49	8.71 + 0.46	8.37 + 0.47
	A	8.59 + 0.27	8.27 + 0.45	8.19 + 0.39

No intra- and inter-groups differences were observed (p > 0.05). Legends: C - cervical; M - middle; A - apical; MC - MaxCem Elite; RU - RelyX U200; GC - GC Gold Label Luting Lining; RL - RelyX Luting 2.

Table 2 displays the mean and standard deviation of tag length formed by the cementation protocols at cervical, middle, and apical thirds of the post-space in the function of the cementation systems. Regardless of the period of storage, inter and intra-group comparisons did not show significant differences (*P* > 0.05).

**Figure 2:**
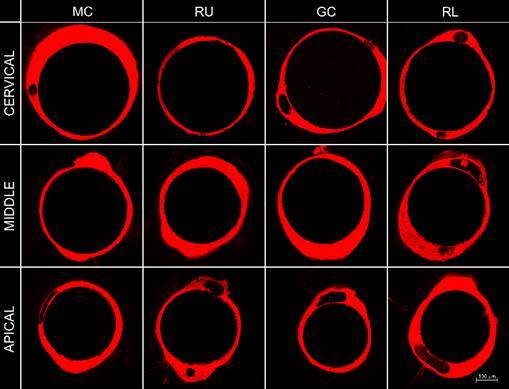
Representative image of tags formation according to the post-space third and cementation system after 24-hour of storage. Abbreviations: MC - MaxCem Elite; RU - RelyX U200; GC - GC Gold Label Luting Lining; RL - RelyX Luting 2. Magnification: 10x; Scale:100 μm.

**Figure 3:**
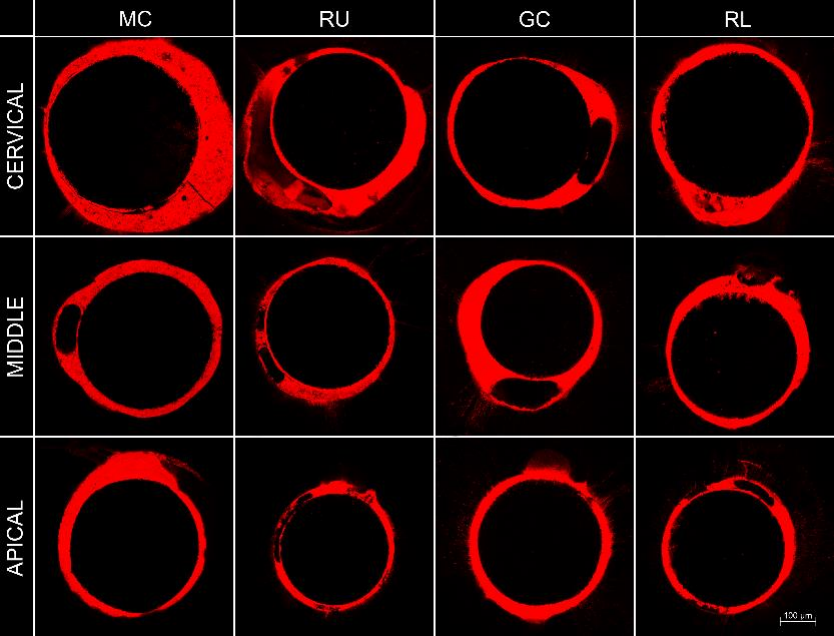
Representative image of tags formation according to the post-space third and cementation system after 6-month of storage. Abbreviations: MC - MaxCem Elite; RU - RelyX U200; GC - GC Gold Label Luting Lining; RL - RelyX Luting 2. Magnification: 10x; Scale:100 μm.

**Figure 4:**
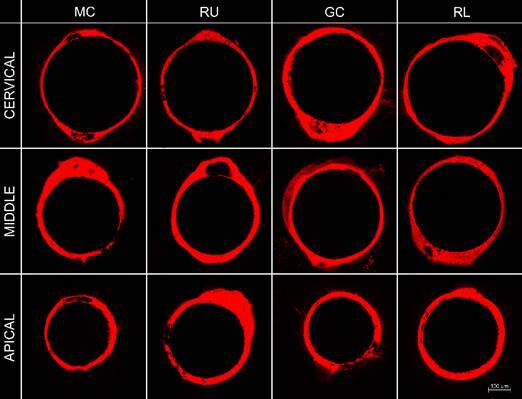
Representative image of tags formation according to the post-space third and cementation system after 12-month of storage. Abbreviations: MC - MaxCem Elite; RU - RelyX U200; GC - GC Gold Label Luting Lining; RL - RelyX Luting 2. Magnification: 10x; Scale:100 μm.

Figures 2, 3, and 4 showed the representative images of the tag’s formation, respectively in 24-hour, 6-month and, 12-month.

## Discussion

This study aimed to evaluate the effect of aging and cementation systems (glass ionomer cement and self-adhesive resin cement) on the bond strength to post-space radicular dentin after GFP cementation. Based on the results, our null hypothesis must be rejected since the cementation systems and period of storage affected the bond strength.

GFP cementation in the root canals is based on the adhesive cementation ^1^. The adhesion between dental materials to radicular dentin is widely evaluated by push-out bond strength tests ^2^ ^,^ ^18^ ^,^ ^20^ ^)^ although it does not fully reproduce the clinical conditions ^5^ ^,^ ^19^ ^,^ ^21^. Some technical characteristics during the push-out test may affect the results, including the materials’ toughness, the placement of the specimen and its relation to the displacement forces, and/or the diameter of the punch or root canals ^21^. The punch diameter should occupy from 50% to 83% of the diameter of the root canal ^21^ ^,^ ^22^. In light of this and to avoid bias in the results, different apical punch diameters were used for each post-space third and only comparisons between the same post-space thirds were performed.

In our study, the sample size was defined based on a pilot study and it agrees with previous studies that used similar methodologies ^5^ ^,^ ^14^ ^,^ ^19^ ^,^ ^23^. All specimens were obtained from bovine teeth since they can reproduce human teeth on bond strength and tags formation studies adequately due to the similarity related to the dentin morphology ^5^ ^,^ ^20^ ^,^ ^21^ ^,^ ^24^.

Based on our results, after 24 hours and 6 months of storage, MC, RU, and GC behave similarly in the cervical and middle thirds in terms of bond strength. However, in the apical post-space third, RU and GC showed higher bond strength than MC and RL. As a glass ionomer cement, GC has a mechanism of adhesion to dentin by chemical bonds between calcium ions from hydroxyapatite and carboxylate groups formed during the acid-base reaction of the material ^3^ ^,^ ^5^, which can explain the better results for GC. Moreover, the similarities in the chemical reactions of the self-adhesive resin cement RU can justify the similar results between RU and GC ^5^. Regarding the post-space thirds, the apical third is hard to reach, hindering an adequate adhesion ^25^, and justifying the lower bond strength values for this third.

Interestingly, we observed that, after 12 months of storage, GC showed the highest bond strength among the cementation systems. This result infers that the chemical bond mechanism of GC is less prone to degradation of the hybrid layer over time. 

The water present in dentin is crucial to maintain the collagen scaffold adequate ^6^ for the infiltration of resinous monomers. However, excessive moisture can separate the phases between the monomers, resulting in poor monomer polymerization ^12^, poor cement infiltration, and gap formation in the bonding interface ^12^. Thus, hydrolytic and enzymatic degradation ^13^ of the hybrid layer, dentin collagen, and/or cementation system can hinder a long-lasting adhesion ^11^ ^)^ and affect inherent characteristics of dentin ^4^ and the retention of GFP in the root canals ^9^.

The bonding of self-adhesive resin cements is dependent on the chemical interaction between the acidic monomers and the hydroxyapatite of the dentin ^7^ ^,^ ^8^ ^,^ ^19^ ^,^ ^26^. Glass ionomer cements and self-adhesive resin cements are less sensitive to the operative technique since they do not require dentin pre-treatment, which infer that their behavior is more material-dependent than technique-dependent ^5^.

A long-lasting adhesion to dentin is also influenced by the water diffusion in the resin-dentin interface as a result of the enzymatic activity of metalloproteinases cysteine and cathepsins from the dentin matrix ^11^ ^,^ ^27^. Our results showed that MC and RU had lower bond strength after 12 months in comparison with GC, which infers that the enzymatic activity can trigger the degradation of the hybrid layer for self-adhesive cementation systems (MC and RU) over time ^6^. However, the cohesive failure mode was the most frequent in all cementation systems, regardless of the evaluation time, possibly due to the chemical composition and the adhesion mechanism of the cementation systems to the root dentin ^5^ ^,^ ^6^ ^,^ ^8^.

The dentinal penetrability can be evaluated by confocal laser scanning microscopy in a non-destructive manner. With this microscopy technique, it is possible to measure the cement infiltration into the dentin both in the dentinal tubules and collagen matrix ^2^ ^,^ ^28^ by using a fluorescence dye. Thus, the rhodamine B dye was incorporated into the cementation systems. Although fluorescence dyes may reduce the monomer’s conversion and the bond strength, the concentration used in this study (0.01%) does not affect the polymerization reaction and bond strength of resin-based materials ^28^ ^,^ ^29^. On the other hand, the effects of the polymerization and/or conversion of monomers on tag formation are uncertain. Therefore, we evaluated the tag formation at all the experimental periods.

The clinical extrapolation of our results must be carefully performed since we have some limitations. Although we made all our efforts, the *in vitro* experimental design is limited and cannot simulate all the conditions of the oral environment. Thus, clinical trials or *in situ* studies are crucial to simulate the natural conditions more reliably manner and to provide strengthening outcomes for clinical decision-making.

Nevertheless, our results highlighted that the bond strength tends to decrease over a year, regardless of the cementation system used. In light of this, it can be inferred that dental clinicians play an important role in the longevity and success rates of the treatment, being as relevant as the material’s properties themselves. Thus, conducting a careful cementation protocol without negligence is crucial to achieving a stable and uniform hybrid layer and a long-lasting bonding. Also based on the results and limitations of our study, the GFP cementation using glass ionomer cement is recommended for increased adhesion to post-space dentin. 

## Conclusion

The bond strength of all cementation systems to post-space dentin decreases over a year. However, the resin tag formation was similar for all the groups. In relation to the type of cement, glass ionomer cement (GC Golden Label Luting & Lining) showed the highest bond strength after 12 months, suggesting adequate clinical performance by a low-cost approach compared to resin cements.
